# Mutualism effectiveness and vertical transmission of symbiotic fungal endophytes in response to host genetic background

**DOI:** 10.1111/j.1752-4571.2012.00261.x

**Published:** 2012-12

**Authors:** Pedro E Gundel, María A Martínez-Ghersa, Marina Omacini, Romina Cuyeu, Elba Pagano, Raúl Ríos, Claudio M Ghersa

**Affiliations:** 1 IFEVA-Facultad de Agronomía (UBA)/CONICETArgentina; 2 MTT Agrifood Research, Plant ProtectionFinland; 3 Instituto de Genética “Ewald A. Favret” Castelar (INTA)Argentina

**Keywords:** environmental stress, genetic specificity, grass–endophyte interaction, hybrid vigor, transmission efficiency

## Abstract

Certain species of the Pooideae subfamily develop stress tolerance and herbivory resistance through symbiosis with vertically transmitted, asexual fungi. This symbiosis is specific, and genetic factors modulate the compatibility between partners. Although gene flow is clearly a fitness trait in allogamous grasses, because it injects hybrid vigor and raw material for evolution, it could reduce compatibility and thus mutualism effectiveness. To explore the importance of host genetic background in modulating the performance of symbiosis, *Lolium multiflorum* plants, infected and noninfected with *Neotyphodium occultans,* were crossed with genetically distant plants of isolines (susceptible and resistant to diclofop-methyl herbicide) bred from two cultivars and exposed to stress. The endophyte improved seedling survival in genotypes susceptible to herbicide, while it had a negative effect on one of the genetically resistant crosses. Mutualism provided resistance to herbivory independently of the host genotype, but this effect vanished under stress. While no endophyte effect was observed on host reproductive success, it was increased by interpopulation plant crosses. Neither gene flow nor herbicide had an important impact on endophyte transmission. Host fitness improvements attributable to gene flow do not appear to result in direct conflict with mutualism while this seems to be an important mechanism for the ecological and contemporary evolution of the symbiotum.

## Introduction

Plant communities of most temperate grasslands around the world have experienced dramatic alterations as a consequence of species invasion and extinction – two processes resulting from human production activities and climate change (Groppe et al. 2001; Thrall et al. 2007). Imminent environmental changes are expected to alter biotic interactions at an unprecedented rate and, as a result, some mutualisms might be lost or turn into parasitic or pathogenic symbiosis (Kiers et al. 2010). Thus, understanding the underlying processes that govern symbiosis resilience is crucial to manage the positive (e.g., nitrogen-fixing bacteria) as well as the negative (e.g., pathogen outbreaks) consequences of such interactions in agro-ecosystems (Thompson 2005; Thrall et al. 2007; Kiers et al. 2010). Cool-season grasses occur throughout continental and maritime temperate regions often associated with leaf fungal endophytes that may locally resist invasions while they may also be threatening invaders as exotics in native communities, old successional fields, and croplands (Clay and Schardl 2002; Vila-Aiub et al. 2003; Saikkonen et al. 2004; Gundel et al. 2009; Rudgers et al. 2009). Despite being a relatively unstudied symbiosis, recent work revealed that grass–endophyte interaction may play a key role in specific grass invasion and extinction processes (Rudgers et al. 2009; Gundel et al. 2009, 2010; Kiers et al. 2010).

Symbiosis between grass species of the Pooideae subfamily and systemic fungal endophytes of the genus *Epichloë* or *Neotyphodium* (i.e., epichloae endophytes; Schardl 2010) is considered to be a defensive mutualism (Clay and Schardl 2002). Both genera produce a set of alkaloids that protect grasses against herbivory and induce eco-physiological changes that make plants more tolerant to different stress factors (Clay and Schardl 2002; Rasmussen et al. 2007; White and Torres 2010). *Epichloë* species are often considered pathogenic to host grasses as they may induce abortion of plant reproductive structures when reaching sexual reproduction state and horizontal spreading, a process known as choke disease (Clay and Schardl 2002; Schardl 2010). Nonetheless, under certain conditions some species of *Epichloë* behave positively on host grass because no stroma is produced (Clay and Schardl 2002; Schardl 2010). Instead, it is well documented that *Neotyphodium* endophytes are hybrids from their relatives *Epichloë* (*Neotyphodium lolii* is an exception; Moon et al. 2000; Selosse and Schardl 2007) that reproduce asexually and spread exclusively vertically from host mother plants to seeds, without symptoms of disease (Clay and Schardl 2002; Saikkonen et al. 2004; Selosse and Schardl 2007; Rudgers et al. 2009). However, it has been shown that *Neotyphodium* endophytes can also depress host fitness when demanding more resources than the benefits they provide (Faeth 2002; Cheplick 2007). Therefore, there is an environment-mediated continuum of interaction types that may range from negative to positive outcomes in the epichloae–grass complex (Clay and Schardl 2002; Saikkonen et al. 2004; Rudgers et al. 2009).

Although mutualisms imply benefits for both partners, the evolutionary stability of this interaction may depend on the long-term balance between costs and benefits (Herre et al. 1999; Saikkonen et al. 2004; Thompson 2005; Kiers et al. 2010). The loss of sexual reproduction and the dependence on vertical transmission may imply evolutionary constraints for the fungal endophyte owing to the inability to generate genetic variability and purge deleterious mutations (Herre et al. 1999; Thompson 2005). Although selection may favor *Neotyphodium* genotypes that are strongly mutualistic for host plants, changes in the effectiveness levels induced by the host plant response to environmental heterogeneous conditions or changes in their genotypes are likely to break down the mutualism stability (Saikkonen et al. 2004; Thompson 2005). In allogamous, wind-pollinated host grasses, genetic conflicts are likely to arise. This is attributed to differential gene flow rates between partners’ populations causing maladaptation or genetic mismatching, a process that has been proposed to destabilize mutualism and explain the loss of infection and variation in infection frequency in wild populations (Saikkonen et al. 2004; Sullivan and Faeth 2004). However, the symbiosis between asexual, vertically transmitted *Neotyphodium* fungi and self-incompatible, wind-pollinated grasses occurs with high level of incidence in wild native and cultivated species worldwide (Gundel et al. 2009; Rudgers et al. 2009). Therefore, how host grasses deal with the maintenance of both genetic variability and symbiosis producing such apparently opposite consequences remains to be accounted for (Gundel et al. 2010).

Many studies have shown that genetic factors modulate the interaction between grasses and epichloae endophytes (Hesse et al. 2003; Hamilton et al. 2010; Saikkonen et al. 2010). Genetic specificity is clear at species level because one host species is usually associated with one fungal endophyte species (Moon et al. 2000). Besides, incompatibility symptoms such as endophyte death, exclusion of all or some of the inoculated fungi from the plant, or deficient vertical transmission have been observed when endophyte hyphae are inoculated into different plants (Christensen 1995; Johnson-Cicalese et al. 2000; Brem and Leuchtmann 2003; Saikkonen et al. 2010). More realistic controlled plant crosses through pollen, although insufficiently explored, have alternatively shown that endophyte activity is affected by the genetic identity of pollen donors (Hiatt and Hill 1997). In addition, the existence of structured populations suggests specificity at population level, where endophytes usually present a reduced number of genotypes associated with genetically variable grass populations (Moon et al. 2000; Arroyo García et al. 2003; Piano et al. 2005; Wäli et al. 2007; van Zijll de Jong et al. 2008; Schardl 2010; Saikkonen et al. 2010). Thus, on the basis of the genetic specificity hypothesis and given the cross-pollination nature of host grasses, genetic variability and symbiosis, two apparent fitness traits for host plants, seem to be under permanent conflict (Saikkonen et al. 2004, 2010; Gundel et al. 2010).

We have previously presented a theoretical model that includes host fitness relationships with inbreeding and outbreeding, and the way in which genotypic variation may affect the expression of the endophyte mutualistic effect on the grass in terms of persistence and productivity (Gundel et al. 2010). This model projects the performance of both partners living in symbiosis (i.e., symbiotum). At low levels of genetic variability in the host population, and if host plants incur energetic costs to maintain the endophyte (e.g., under resource-shortage; Faeth 2002; Cheplick 2007), a negative parasitic effect is expected from the host inbreeding depression, despite the high endophyte compatibility (Gundel et al. 2010). At intermediate level of host genetic variability, the mutualistic benefits should be at their highest potential and infected plants would have higher fitness than noninfected ones. At these levels of genetic variability (within the population), both plant fitness (regardless of infection status) and mutualism effectiveness increase as genetic distance between mating parents is greater (Ellstrand and Schierenbeck 2000; Gundel et al. 2010). The increase in host plant fitness associated with heterosis (i.e., hybrid vigor) may reduce both the importance of the mutualistic benefit and the cost of plant infection, and thus the endophyte could behave as a free rider. Finally, at high levels of host genetic variability, compatibility mismatch would reduce endophyte-infected plants fitness regardless of the pollen source (distant genotypes within species or genetically related plant species). On the other hand, crossing between individuals of genetically distant plant populations would express overall low fitness because of outbreeding depression (Ellstrand and Schierenbeck 2000; Gundel et al. 2010).

**Figure 1:**
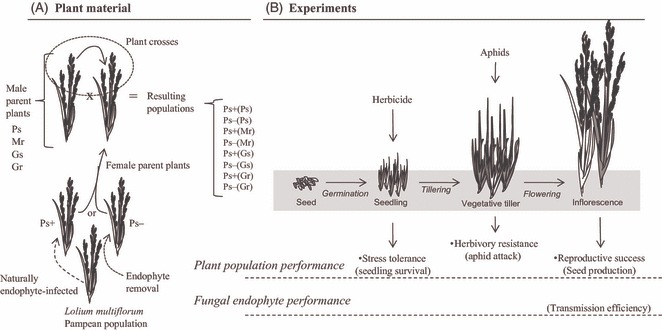
Schematic diagram depicting the generation of the plant material used for the experiments. (A) Endophyte was removed from the *Lolium multiflorum* Pampean population naturally infected with *Neotyphodium occultans* endophyte to obtain female parent plants from the same population with contrasting infection level (Ps+ and Ps−). Plants from these populations were crossed with male parent plants from four different populations and the resulting ones were Ps+(Ps), Ps−(Ps), Ps+(Mr), Ps−(Mr), Ps+(Gs), Ps−(Gs), Ps+(Gr), and Ps−(Gr). (B) Response variables used to estimate the performance of both *host plant population* and *endophyte symbiont* subjected to different stresses during the grass life cycle.

From this perspective, the objective of this study is to explore the importance of host genetic background in modulating the performance of both host plant and fungal endophyte as an integrated unit. We propose that gene flow by pollen, as it naturally occurs, is a fitness trait because it benefits the host plant population by injecting hybrid vigor and provides raw material to the symbiotum for contemporary evolution. We manipulated the infection status in a *Lolium multiflorum* host population naturally infected with fungal endophyte *Neotyphodium occultans*, used as reference population, and then, we performed intra- and interpopulation plant controlled crosses to change the host genetic background and thus the potential genetic specificity between grass and fungus populations (Fig. 1). Seedling survival to herbicide, aphid attack, and seed production in the resulting F1 plants were evaluated as surrogate variables of stress tolerance, herbivory resistance, and reproductive success. In addition, the efficiency of vertical transmission from plant to seeds is evaluated as a measure of endophyte performance (Fig. 1). We predict a positive effect of endophyte on plant tolerance to stress, herbivory resistance and seed production, and also that these effects (except for herbivory resistance) will be overcome by the presence of specific herbicide resistance genes and hybrid vigor in interpopulation hybrids (Gundel et al. 2010). The endophyte and host genetic background interaction effects are expected to impact on the endophyte transmission process. We predict a reduction in transmission efficiency because of the addition of new genes and intrapopulation genetic variability.

## Materials and methods

### Plant material and study model

We manipulated the endophyte infection status and host genetic background of *Lolium multiflorum* plants from a naturalized population with natural high level of *Neotyphodium occultans* endophyte collected in Inland Pampa subregion (Argentina). As endophyte is transmitted by host seed and not by pollen (Siegel et al. 1984), endophyte removal was achieved by treating mature endophyte-infected seeds collected in December 1998, with a systemic fungicide (triadimenol, see details in Vila-Aiub et al. 2005). Thus, seeds treated and nontreated with fungicide were cultivated on 1 m^2^ plots in the experimental field at School of Agronomy, University of Buenos Aires (34°35′S, 58°35′W), for obtaining two populations with high (Ps+) and low (Ps−) endophyte infection level within the original Pampean population. Plants from both populations were annually cultivated in adjacent plots to allow pollen exchange and to prevent genetic segregation.

In 2003, plants of other *L. multiflorum* populations were also sown in plots in the same experimental garden for the plant crosses (Fig. 1; Table 1). Two forage commercial varieties without fungal endophytes were included: Marshall and Gulf (Redfearn et al. 2002). From these two herbicide-susceptible varieties (Ms− and Gs−), herbicide-resistant isolines (Mr− and Gr−) were obtained by applying higher doses of diclofop-methyl herbicide and successive backcrosses (R. E. Baker, personal communication). More than 1000 plants from each population were grown in 1 m^2^ plot, and each plot was covered at flowering, by 2-m plastic film wall to prevent pollen exchange among plants from different populations. Mature seeds were hand-harvested, threshed, and stored in dark glass jars at 10°C. Infection level was determined in each population by evaluating endophyte presence in 100 seeds. Seeds were incubated for ≍12 h in NaOH (5%) and stained with Bengal Rose stain to look for the typical *N. occultans* hyphae under light microscope (Bacon and White 1994; Moon et al. 2000). Detailed information of each population is given in Table 1.

**Table 1:** Diclofop-methyl herbicide resistance level and *Neotyphodium occultans* endophyte infection of the *Lolium multiflorum* populations used for the plant crosses. Name of each population (nomenclature) stands for the first letter of original population, level of resistance (r: resistant or s: susceptible) and infection level (+ ≥95 or −≤5%)

Original population	Herbicide resistance	Endophyte infection (%)*	Nomenclature
Pampean	Susceptible	95	Ps+
	Susceptible	5	Ps−
Marshall	Susceptible	0	Ms−
	Resistant	0	Mr−
Gulf	Susceptible	0	Gs−
	Resistant	0	Gr−

* Based on 100 examined seeds per population under microscope.

### Genotypic characterization of each population and plant crosses

Each plant population was genetically characterized through nuclear micro-satellites (SSR, single sequence repeat). 45 micro-satellites were used to detect polymorphic variability among populations. First, 38 and 40 individual plants were analyzed for Ps+ and Ps− populations, respectively. Difference between populations was assessed by comparing the genetic variability among plants within versus between populations, and the formal statistical test was performed through molecular analysis of variance (amova; Excoffier et al. 1992). Besides, the information provided by each SSR (presence/absence) was assessed by means of principal coordinates analysis (PCO). Second, a similar number of plants were used for the analysis of the other four populations (Ms−, Mr−, Gs−, and Gr−), but in this case, all the plants were bulked. Finally, the polymorphic information of the six *L. multiflorum* populations used for the plant crosses (Fig. 1) was compared through a cluster analysis (UPGMA) that estimate the genetic distances among them through Jaccard’s coefficient. Analyses were performed with NTSYS-pc, Numerical Taxonomy System (2.01) (Rohlf 1998).

Because *L. multiflorum* is self-incompatible, all the seeds harvested in a plant are produced by mother’s ovules fertilized by different parent’s pollens. Thus, we changed the genotype in F1 seeds by controlling parent plants (Fig. 1). One hundred plants of Ps+ and Ps−, and 50 plants of Ms−, Mr−, Gs−, and Gr− were grown during the normal growing season (autumn–winter–spring) in 2004. Individual plant seeds were directly sown in 1.5-L pots, filled with a soil mixture of organic black soil, sand and peat-moss (50, 25, and 25%, *v*/*v*). Plants were periodically irrigated. On the basis of the flowering synchrony, two plants (one as female parent plant and other as male parent plant) were selected for the experimental crosses. Each pair of plants was covered by a wax paper bag hung from a wire to control each cross and to also prevent pollen contamination.

Ps+ and Ps− plants from the Pampean population were used as female parent plants, while plants from that populations (either Ps+ or Ps−) and Ms−, Mr−, Gs−, and Gr− were used as male parent plants (Fig. 1). Ten plants from each Ps+ and Ps− population were isolated to have a measure of the self-incompatibility degree and pollen contamination. Resistance to herbicide in F1 hybrid plants of resistant isolines (Mr− or Gr−) is evidence that the crossing plant method has worked because it indicates that the resistance genes has been transferred from the male parent plant to the progeny (see Results). The plants were harvested and threshed at the end of the growing cycle. Seeds produced by each type of plant cross were counted and tested for endophyte infection. Isolated plants [Ps+(self) and Ps−(self)] and F1 hybrid plants of Ps+(Ms−) and Ps−(Ms−) were not included in the experiments because of a lack of enough seed or problems in their level of infection (Table 2). Resulting populations were inbred populations [Ps+(Ps) and Ps−(Ps)] and F1 interpopulation hybrids (Fig. 1; Table 2).

**Table 2:** Seed production and level of endophyte infection of *Lolium multiflorum* plant populations obtained after different crosses among the original populations

Plant crosses					
			
Female parent plants	Male parent plants	Resulting populations	Number of crosses	Number of seeds produced	Endophyte infection (%)*
Ps+	None	Ps+(self)	10	3	No data
Ps−	None	Ps−(self)	10	2	No data
Ps+	Ps	Ps+(Ps)	21	396	95
Ps−	Ps	Ps−(Ps)	20	240	5
Ps+	Ms	Ps+(Ms)	12	188	50
Ps−	Ms	Ps−(Ms)	9	158	10
Ps+	Mr	Ps+(Mr)	14	56	95
Ps−	Mr	Ps−(Mr)	8	67	5
Ps+	Gs	Ps+(Gs)	13	203	100
Ps−	Gs	Ps−(Gs)	6	141	5
Ps+	Gr	Ps+(Gr)	14	262	95
Ps−	Gr	Ps−(Gr)	7	121	0

* Based on 20 examined seeds per population under microscope.

### Performance of plant populations

Two bioassays of herbicide dose response were carried out at seedling stage (Fig. 1). Plants were subjected to a gradient of sublethal herbicide doses of diclofop-methyl (i.e., factor of oxidative stress), a pre-emergent herbicide used to control grass weeds in winter crops (Martínez-Ghersa et al. 2004). The mechanism of action is the inhibition of the acetyl-coenzyme A carboxylase enzyme, key in the pathway of lipid biosynthesis, and it is coded by a partial-dominant single gene (Betts et al. 1992; Powles and Holtum 1994). Heterozygous plants show intermediate resistance level at recommended doses (Betts et al. 1992). The disruption of membrane potential, disturbance of proton gradient, and oxidative stress are all symptoms caused by the second mechanism of action that has been described for the herbicide (Powles and Holtum 1994).

In the first experiment (Exp. 1), effects of endophyte infection and host genetic background on plant tolerance to different herbicide doses were evaluated by comparing seedling survival of Ps+(Ps), Ps−(Ps), Ps+(Mr), and Ps−(Mr), while in the second experiment (Exp. 2), the evaluation was carried out on seedlings of Ps+(Ps), Ps−(Ps), Ps+(Gs), Ps−(Gs), Ps+(Gr), and Ps−(Gr). In May 2005, eight seeds per population were sown in pots (1.5 L for the Exp. 1, and 25 L for the Exp. 2) filled with the same substrate as above. The bioassays were carried out outdoors in the same experimental field. Within each bioassay, the pots were at random arranged and they were irrigated according to demand. Three different herbicide doses [0 (control), 70 and 140 g of active ingredient (ai) per ha (label dose: 560 g ai ha^−1^)] were sprayed on 2–3 leaves seedlings using a constant-pressure hand-sprayer of 1 L (Commercial formulation 284 g ai L^−1^, Iloxan; Hoechst-Aventis). Three replicate pots were used for each treatment combination. Alive seedlings were recorded in each pot one month after herbicide application.

The presence and size of bird cherry-oat aphid colonies (*Rhopalosiphum padi* L.) that naturally established and fed on *L. multiflorum* plants were measured in the Exp 1. Although fungal alkaloids were not measured, it is well known that endophyte-infected *L. multiflorum* plants present peramine and lolines that have active toxic effects against insects (TePaske et al. 1993; Omacini et al. 2009). During plants’ vegetative tiller stage (Fig. 1), the frequency of tillers with aphids per pot and the number of aphids per tiller (based on 10 tillers per pot) were recorded.

Because our goal was to study the endophyte effect on plant performance in interaction with host genetic background, and considering the great potential for expressing hybrid vigor in F1 interpopulation hybrids typical for self-incompatible species, we compared the relative variation in seed production in both experiments (Fig. 1). Given that the herbicide had an important effect on plant density affecting the individual plant yield, the comparison was only done in the control situation for both experiments (see Appendix 1 for complete analysis). In experiment 2, only Ps+(Ps), Ps−(Ps), Ps+(Gs) and Ps+(Gr) were included. Every spike was harvested at maturity, and after threshing, the number of the total seed produced per spike was obtained. Total seed produced per pot was obtained by the sum all the seeds per spike, all the spikes per plant, and all the plant per pot.

### Performance of endophyte symbiont

Endophyte transmission efficiency, a process likely sensitive to the host genetic background and environmental conditions, is one measure of the fungus performance (Gundel et al. 2008, 2010; Saikkonen et al. 2010). Seed infection was evaluated using the same technique as before. This technique is suitable for endophyte detection in seeds because the seed is the plant structure with the highest hyphae concentration, and, in particular, the conspicuous morphology of *N. occultans* makes recognition and evaluation easy to perform (Moon et al. 2000). Ten or all the seeds (if the spike produced <10 seeds) were evaluated per spike in all the spikes per plant. The endophyte transmission efficiency per plant was estimated dividing the sum of all infected seeds by the total evaluated seeds of a plant. Transmission efficiency per pot was estimated by averaging the endophyte transmission efficiency of three to six randomly selected plants in the experiment 1 and about three plants in the experiment 2.

### Data analyses

Seedling survival to herbicide (stress tolerance), aphid attack (herbivory resistance), and endophyte transmission efficiency were analyzed with generalized linear models (glm function, r software, version 2.14.0; R Development Core Team 2011). For both experiment, the effects of endophyte infection, host genetic background, and herbicide dose on the proportion of seedling survival were analyzed considering the response variable as binomial (seedling alive or dead) and using a logit link function. Similarly, endophyte transmission efficiency (proportion of endophyte-infected seeds per pot) as affected by host genetic background and herbicide dose was analyzed for the endophyte-infected crosses. Proportion of tillers with aphids per pot and colony size (number of aphids per tiller) as affected by the endophyte, host genotype, and herbicide dose was analyzed using Poisson distribution and log link function only for experiment 1. In all the cases, minimum adequate models were obtained by removing nonsignificant terms from every maximal model (*P* > 0.05). Stepwise model simplification started by removing the most complex interactions, one at a time, to the simplest one, and *F*-tests or chi-squared tests were run to assess the significance of the increase in deviance that may result by removing a term from a model (Crawley 2007; Zuur et al. 2007). When necessary, overdispersion was compensated. Analyses of deviance were performed to estimate the effect of each factor in the selected models.

Because we wanted to evaluate the endophyte impact in relation to host genetic background, specifically hybrid vigor promoted by gene flow, relative reproductive success was evaluated only in control situations (without herbicide) where plant density was not modified. Relative reproductive success was calculated as the subtraction of seed production per plant for each population cross to the seed production per plant of reference population [Ps+(Ps)]. Statistical analyses were carried out separately for each experiment by using general linear models (lm function, r software, version 2.14.0; R Development Core Team 2011), using the same principle of model selection as before. Assumptions of normality and homogeneity of variance were met without data transformation. The interaction between endophyte infection status and host genetic background was only estimated for experiment 1, while the effect of cross-populations was estimated for experiment 2. In this latter case, Tukey’s honest significant difference was used to pair-compare each cross (Tukey-HSD). Finally, the global statistical analyses evaluating the effect of endophyte infection, host genetic background, and herbicide on seed production per pot is provided in Appendix 1.

## Results

### Genetic distance of population crosses

**Figure 2:**
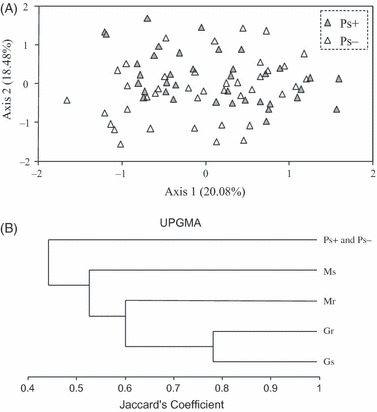
Principal coordinate analysis (A) made with 38 and 40 *Lolium multiflorum* plants from the Pampean populations Ps+ and Ps−, respectively (the proportion of the variance explained by each axis is indicated between parentheses). Cluster analysis (B) built with the UPGMA algorism that estimates the genetic distance among *L. multiflorum* parent populations (Ps+, Ps−, Ms, Mr, Gs, and Gr).

The SSR-based genetic analysis did not detect the differences between plants from the Ps+ and Ps− populations (*P* = 0.360), indicating that the genetic variability within is as high as between populations. This indicates that 5 years since the endophyte was removed, and annual cultivation of both populations in adjacent plots produced no genetic segregation between them (Fig. 2). On the other hand, the Pampean populations were genetically different from the commercial varieties (Fig. 2). Gulf was the most genetically distant population, independently of the isoline (Gs or Gr). Marshall showed an intermediate distance between Pampean and Gulf; Marshall isolines showed some genetic difference between them (Fig. 2).

**Figure 3:**
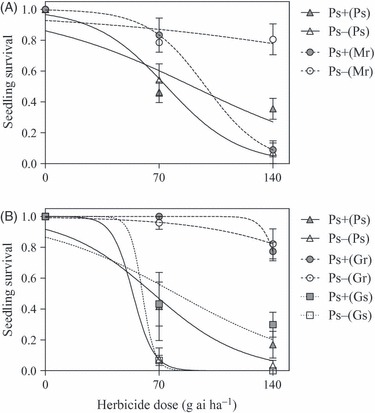
Seedling survival calculated as the proportion of alive seedlings one month after the application of different doses of diclofop-methyl herbicide (g active ingredient per ha) of each *Lolium multiflorum* cross resulting from the combination of endophyte infection and host genetic background in the experiment 1 (A) and 2 (B). Values are means ± SE (*n* = 3). Lines correspond to the modeled logistic curve response of each population.

*Lolium multiflorum* plants from the Pampean population were highly self-incompatible with only five seeds produced by 10 Ps+(self) and 10 Ps−(self) isolated plants (Table 2). In addition, the expression of herbicide resistance in F1 hybrid plants whose male parents were resistant (Mr− or Gr−) is evidence that the system of plant crosses worked well (Fig. 3).

### Plant population performance

The proportion of seedling survival in response to herbicide dose in the experiment 1 depended on the three-way interaction between endophyte infection, host genetic background, and herbicide dose (*F*_2,24_ = 15.36, *P* < 0.001). While hybridizing *L. multiflorum* Pampean plants with plants that belong to the Marshall resistant isoline enhanced seedling survival independently of their endophyte infection level at the intermediate herbicide dose, the endophyte infection reduced seedling survival of the Ps+(Mr) hybrid plants at the highest dose. At the same herbicide dose, however, the endophyte increased seedling survival in the Pampean population (Fig. 3).

The proportion of seedling survival in response to the herbicide dose in the experiment 2 depended on the two-way interaction between endophyte infection and host genetic background (*F*_2,46_ = 4.74, *P* = 0.014), and on the main effect of herbicide (*F*_2,48_ = 138.60, *P* < 0.001). In this case, endophyte infection did not modify the already high survival in the hybrid seedlings between Pampean and Gulf resistant isoline [Ps+(Gr) and Ps−(Gr)], but it increased survival in hybrid seedlings between Pampean and Gulf susceptible isoline [Ps+(Gs) and Ps−(Gs)]. Similarly, the same pattern of endophyte positive effect on survival was observed for the Pampean seedlings. As a result, Ps+(Ps) and Ps+(Gs) were more tolerant than Ps−(Ps) and Ps−(Gs) (Fig. 3).

**Figure 4:**
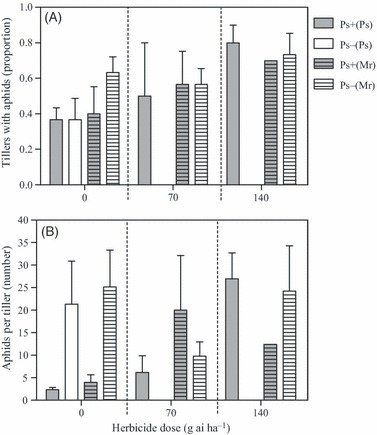
Proportion of tillers per plant colonized by spontaneous aphids *Rhopalosiphum padi* (A) and number of aphids per tiller (B) in relation to the sprayed doses of diclopfop-methyl herbicide (g active ingredient per ha) on the *Lolium multiflorum* plant populations. Values are means ± SE (*n* = 3).

The proportion of tillers with aphids was independent on the tillers per pot (*F*_1,24_ = 0.125, *P* =0.727) but was found to be dependent on the herbicide dose (*F*_1,24_ = 8.53, *P* = 0.007) (Fig. 4). The number of aphids on colonized tillers (colony size) showed also a positive relationship with herbicide dose (*F*_1,22_ = 5.53, *P* = 0.029) (Fig. 4). In the control situation, endophyte strongly decreased aphid infection (*F*_1,10_ = 20.16, *P* = 0.002) independently of host genetic background (*F*_1,8_ = 0.05, *P* = 0.826).

**Figure 5:**
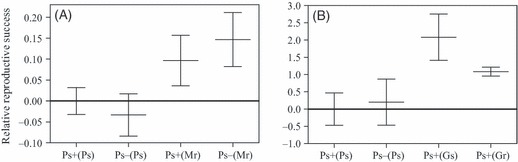
Relative productive success calculated as the subtraction of seed production per plant of each population cross [experiment 1: Ps−(Ps), Ps−(Mr), and Ps−(Mr); and experiment 2: Ps−(Ps), Ps+(Gs), and Ps+(Gr)] to the seed production per plant of reference population [Ps+(Ps)] under control situation (without herbicide). Values are means ± SE (*n* = 3).

In general, the reproductive success of plants was not affected by the endophyte under the control situations (without herbicide). In experiment 1, seed production per plant depended only on the host genetic background (*F*_1_ = 6.99, *P* = 0.029), independently of the endophyte infection status (*F*_1_ = 0.59, *P* = 0.463) (Fig. 5). The F1 interpopulation hybrids produced ≍2.00 g of seeds per plant (≍1042 seeds) in the control pots meanwhile the F1 intrapopulation hybrids produced 1.15 g (≍577 seeds) (see Appendix 1). In experiment 2, the relative reproductive success was affected by the population indicating that at least one of the crosses differed in seed production relative to the reference populations (*F*_1_ = 10.19, *P* = 0.004). The only significant difference (Tukey-HSD) was between Ps+(Ps) and Ps+(Gs) (*P* = 0.004) (Fig. 5). On average, the Ps+(Gs) populations produced more than twofold seeds (≍34.28 g; ≍17 140 seeds) that the F1 intrapopulation hybrids infected and noninfected (≍19.72 g; ≍9860 seeds), while Ps+(Gr) showed an intermediate value (≍ 26.32 g seeds; ≍13 160 seeds) (see Appendix 1).

### Performance of endophyte symbiont

In experiment 1, neither the host genetic background (*F*_1,48_ = 1.46, *P* = 0.232) nor the herbicide dose (*F*_1,47_ = 0.05, *P* = 0.815) was found to affect the transmission efficiency of the endophyte (Table 3). Fifty-five plants were analyzed in this experiment, and 52% of them had perfect transmission (i.e., all the seeds were endophyte-infected). Twenty-five percent of the plants presented transmission efficiency between 0.99 and 0.80, while 23% of the plants presented an endophyte transmission efficiency lower than 0.80. In experiment 2, transmission efficiency was not affected by either the two-way interaction between both factors (*F*_2,21_ = 2.45, *P* = 0.110) or by the herbicide alone (*F*_1,23_ = 0.01, *P* = 0.927; Table 3) while there was an effect associated with the host genetic background, with Ps+(Gs) presenting higher transmission than the other two (*F*_2,24_ = 5.45, *P* = 0.012; Table 3). Of the sixty plants analyzed in experiment 2, 60% of the plants had a perfect transmission, 23% presented transmission efficiency between 0.99 and 0.80, and only 17% of the remaining plants presented endophyte transmission efficiency lower than 0.80.

**Table 3:** Efficiency of endophyte vertical transmission (i.e., proportion of infected seeds) in the different *Lolium multiflorum* population crosses exposed to different doses of diclofop-methyl herbicide (g ai ha^−1^). Values are means and SE between parentheses (*n* = 3) from both experiments (1 and 2)

	Herbicide dose		
	
Population cross	0	70	140
Experiment 1			
Ps+(Ps)	0.86 (0.07)	0.97 (0.02)	1 (0)
Ps+(Mr)	0.94 (0.03)	0.97 (0.02)	0.84 (0.01)
Experiment 2			
Ps+(Ps)	0.84 (0.15)	1 (0)	0.78 (0.24)
Ps+(Gs)	1 (0)	0.90 (0.15)	0.99 (0)
Ps+(Gr)	0.68 (0.24)	0.84 (0.20)	0.94 (0.09)

## Discussion

Our results provide experimental demonstration that plant genetic background interacts with endophyte symbiont, which affects the performance of the symbiotum, determining the response of the *L. multiflorum* population to stress. The control of the host genetic background on the expression of mutualism changed with the type and level of environmental stress, becoming important only in the response to some doses of herbicide. In early stages of plant cycles, endophyte infection improved seedling survival of susceptible genotypes to abiotic stress (herbicide) and negatively affected the aphid colonies that feed on symbiotic plants, in accordance with previous studies (Omacini et al. 2001; Vila-Aiub et al. 2003). However, while genetic background controlled the endophyte effect on herbicide tolerance, it showed no significant differences on resistance to herbivory. It is noteworthy that seedling survival was negatively affected by the endophyte at the highest herbicide dose (140 g ai ha^−1^) in the F1 interpopulation hybrids only with Marshall resistant isoline but not with Gulf resistant isoline. Besides, although the endophyte conferred resistance to aphids for the two tested host genetic backgrounds in the control situation, this effect was completely eliminated by the herbicide action even at the lowest dose (70 g ai ha^−1^). Considering that endophytes can be costly for host plants under restrictive growth conditions (nutrient or water limitation; Faeth 2002; Cheplick 2007), endophyte benefits can be overwhelming under stressful situations. However, the definite outcome will depend on the relative importance of the different selection pressures (abiotic stress versus herbivory) operating on infected and noninfected plants in the populations.

**Figure 6:**
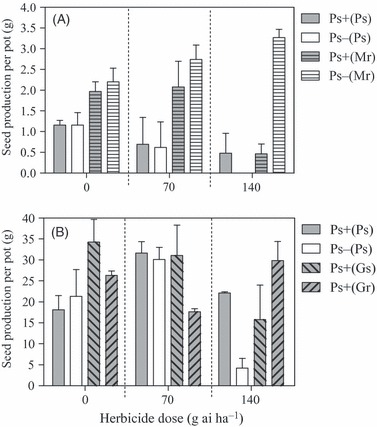
Total production of seeds per pot (g) of *L. multiflorum* plants from the different population crosses in relation to the herbicide dose of diclofop-methyl (g active ingredient per ha) for population crosses Ps+(Ps), Ps−(Ps), Ps+(Mr), and Ps−(Mr) in experiment 1 (A) and for Ps+(Ps), Ps−(Ps), Ps+(Gs), and Ps+(Gr) in experiment 2 (B). Values are mean ± SE (*n* = 3).

Total seed production is the most important parameter of reproductive success in annual plant species (Elam et al. 2007; Radosevich et al. 2007). Because plant density in control situation was the same among populations within each experiment, relative seed production per plant used in this study is equivalent to population yield. Contrary to our prediction and despite the fact that infected *L. multiflorum* plants have been found to produce more seeds than their noninfected counterpart, when growing without plant competition (Vila-Aiub et al. 2005), the fungal endophyte was not associated with higher seed production in our study. Although we have not measured evenness within populations, the nondifferential yield of infected and noninfected plants suggests similar intraspecific competition (at least for the plant densities explored here). In addition, there was no clear difference between infected and noninfected plants as to their ability to offset the reduction in density resulting from the herbicide action. In fact, only noninfected hybrid plants were able to over-compensate reductions in plant density. Alternatively, surviving noninfected plants in experiment 2 [Ps−(Ps)] were able to fully compensate yield at the intermediate but not at the highest herbicide dose (see Appendix 1, Fig. 6). In turn, the unexpected low tolerance of infected hybrid plants [Ps+(Mr)] to the highest herbicide dose was only partially compensated in terms of plant yield (see Appendix 1). In summary, seed production of *L. multiflorum* populations was for some conditions, higher and more stable in the presence of mutualism, not because of a compensation capacity but because of the higher survival to herbicide.

On the other hand, host genetic background and the presence of specific resistant genes increased ecological fitness in *L. multiflorum* populations (measured in terms of reproductive success). Seed production per pot can be the result of combining ability or hybrid vigor between plants from different populations (Elam et al. 2007). Therefore, heterosis degree should be higher, thus conferring fitness to those plants resulting from crosses between genetically distant populations (Ellstrand and Schierenbeck 2000; Radosevich et al. 2007). The cluster analysis suggests that genetic distance among crossed plant populations is higher between Pampean and Gulf, and intermediate between Pampean and Marshall. Although it is not possible to establish a direct relationship between genetic distance (≍ level of heterosis) and seed production per pot, our relative reproductive success analysis for control situations (without herbicide) clearly shows that crosses between genetically distant plants presented a higher yield than the inbred reference population. Provided that endophyte transmission efficiency has not been affected by changing the host genetic background, the frequency of endophyte-infected plants would be higher because of the improved seed production, as a result of combining ability of genotypes or hybrid vigor in host populations.

Most studies on grass–epichloae endophyte symbiosis have focused on the effects of endophyte on host plants rather than on the performance of said symbiosis as an integrated system (Gundel et al. 2008, 2010). Thus, endophyte performance has been scarcely considered. Recent studies have shown that efficiency of endophyte transmission from plant to seeds can be important under natural conditions, and it was suggested that variations in such a trait could depend on the populations and/or the environment (Gundel et al. 2009; Rudgers et al. 2009). However, neither of these factors (i.e., genotype or environment) has been controlled yet. In our study, neither host genetic background nor the environmental stress level strongly affected transmission efficiency. Although the compatibility selection process can continue after the seed stage, our results suggest that vertically transmitted fungal endophytes may be generalist within species, presenting high plasticity to the changes in host plant phenotype (Saikkonen et al. 2010; Gundel et al. 2010). Overall, endophyte transmission efficiency was very high as 61% of 115 plants analyzed showed perfect transmission (1.00), and only 9% presented transmission efficiency lower than or equal to 0.5.

Given the unprecedented environmental changes driven by human activities and climate change, the stability and persistence of symbiotic interactions will depend on the life history traits that control the partners relative rate of evolution in the community context (Thrall et al. 2007; Kiers et al. 2010). Sufficient heritable genetic variation is essential for evolutionary adaptation in response to environmental change. Founder populations are small, and heritable genetic variation is expected to be low during a colonization phase. In addition, dramatic reductions in heritable genetic variation in plant populations are expected after population size decreases because of extreme events such as drought, flooding, fire or herbicide treatment, and as a consequence of habitat fragmentation (Ellstrand and Schierenbeck 2000; Groppe et al. 2001; Elam et al. 2007; Radosevich et al. 2007). For this reason, the evolutionary problem for a successful invasion or an extinction failure to proceed is strongly related to how the species maintains or accumulates heritable genetic variance. This is the raw material for populations to evolve by acquiring adaptive traits assuring fitness to particular environments. The association with endophyte may improve the host invisibility by increasing the tolerance to stresses and maintaining a high population size while, alternatively, it may be costly for fitness depressed plants, because of inbreeding. However, interspecific or intraspecific hybridization of plant populations can diminish the loss of additive genetic variance during founder or fragmentation events and generate novel genotypes. Progressive increase in progeny fitness is expected with greater genetic distance between mating parents. This relationship results from the reduction of inbreeding depression caused by the increment in heterosis and hybrid vigor (Ellstrand and Schierenbeck 2000; Elam et al. 2007; Radosevich et al. 2007). However, if parental genetic distance is too large, progeny fitness is reduced as a result of outbreeding depression. Thus, at intermediate genetic distance among plant mating parents, progeny fitness relative to one of the parents is maximized.

Symbiotic fungal endophytes influence grass adaptive responses to environmental change by altering the loss of heritable genetic variance attributable to biotic or abiotic selection pressures (Hesse et al. 2003; Vila-Aiub et al. 2003; Gundel et al. 2010). Considering that the symbiotic phenotype is an emergent property from the action of both partners (Johnson et al. 1997), our results suggest that the endophyte success is not jeopardized by the fitness gain resulting from hybrid populations. Even though gene flow may increase genetic mismatches between partners, it may be highly compensated by endowing the symbiotum with a mechanism for contemporary evolution (Saikkonen et al. 2004, 2010; Gundel et al. 2010). As it has been pointed out for symbiosis between aphids and their endosymbionts, the higher rate of evolution in one of partners may increase mutualism stability, by adapting the symbiotum to environmental changes (Kiers et al. 2010). Under this perspective, the higher gene flow rate of host grasses and their consequent high genetic variability would constitute an essential mechanism for the ecological resilience and contemporary evolution of the symbiotum (Saikkonen et al. 2004; Gundel et al. 2010).

## Data archiving statement

Data deposited in the Dryad repository: doi: 10.5061/dryad.d6n271pv.

## Appendix 1

The global analysis of variance for seed production per pot in the experiment 1 (Fig. 6A) showed a marginal two-way interaction between endophyte infection status and herbicide dose (*P* = 0.055), which corresponded with no differences between infected and noninfected counterparts except at the highest dose (140 g ai ha^−1^). In addition, there was a significant effect of host genetic background (*P* = 0.003) because those plants whose parents were genetically resistant (Mr) presented on average a higher production of seeds at all doses (no interaction effect, *P* = 0.205). Although seed production of Ps+(Mr) was clearly lower than Ps−(Mr) at the highest dose, the effect of host genetic background was found independent on the infection status (*P* = 0.134) (Table A1).

**Table A1:** Analyses of variance for the total production of seeds per pot (g) of *Lolium multiflorum* plants as affected by endophyte infection status, host genetic background, herbicide dose and their interactions in the case of experiment 1, and as affected by the population cross, herbicide dose, and their interaction in the case of experiment 2

Factor	DF	*F*-value	*P*-value
Experiment 1			
Endophyte infection status (E)	1	11.266	**0.004**
Host genetic background (G)	1	12.602	**0.003**
Herbicide dose (H)	2	0.619	0.551
E × G	1	2.494	0.134
E × H	2	3.482	**0.055**
G × H	2	1.754	0.205
E × G × H	1	0.102	0.753
Experiment 2			
Cross (C)	3	1.888	0.158
Herbicide dose (H)	2	4.880	**0.017**
C × H	6	4.256	**0.005**

Bold values indicate significant or marginally significant effects (*P* < 0.05).

In the experiment 2, the production of seeds per pot of the population crosses interacted with the dose of herbicide (*P* = 0.005) (Table A1). In spite that it is not possible to estimate the effect of the endophyte infection status, the three endophyte-infected crosses [Ps+(Ps), Ps+(Gs), and Ps+(Gr)] were able to present a higher seed production relative to the noninfected cross [Ps−(Ps)] at the highest herbicide dose (Fig. 6B).
